# Growth, clinical and neurodevelopmental outcomes at school age are similar for children who received 1-year lamivudine or lopinavir/ritonavir HIV prophylaxis in early life

**DOI:** 10.1038/s41598-021-82762-8

**Published:** 2021-02-04

**Authors:** Nicolas Nagot, Mandisa Singata-Madliki, Amandine Cournil, Joyce Nalugya, Souleymane Tassembedo, Catherine Quillet, Melany W. Tonga, James Tumwine, Nicolas Meda, Chipepo Kankasa, Mwiya Mwiya, Paul Bangirana, Marianne Peries, Joanne Batting, Ingunn M. S. Engebretsen, Thorkild Tylleskär, Philippe Vande Perre, Grace Ndeezi, Jean-Pierre Molès

**Affiliations:** 1 Pathogenesis and Control of Chronic and Emerging Infections, INSERM U1058, Université de Montpellier, Etablissement Français du Sang, Université des Antilles, 60, rue de Navacelles, 34394 Montpellier Cedex, France; 2 University of Fort Hare, East London, South Africa; 3 School of Medicine, College of Health Sciences, Makerere University, Kampala, Uganda; 4 Centre MURAZ, Bobo-Dioulasso, Burkina Faso; 5 Department of Paediatrics and Child Health, University Teaching Hospital, Lusaka, Zambia; 6 Centre for International Health, University of Bergen, Bergen, Norway

**Keywords:** HIV infections, Paediatric research

## Abstract

In the ANRS 12174 trial, HIV-exposed uninfected African neonates who received lopinavir-ritonavir (LPV/r) prophylaxis for 1 year exhibited slower growth from birth to week 50 compared with those receiving lamivudine (3TC). We assessed whether this difference in growth persisted over time, and was accompanied by differences in neuropsychological and clinical outcomes. Between February 2017 and February 2018, we conducted a cross-sectional clinical evaluation among former trial participants who completed the 50-week follow-up and who were not HIV-infected. In addition to clinical examination, neuropsychological outcomes were assessed using the tests Kaufman-ABCII, Test of Variables of Attention, Movement Assessment Battery for Children and the Strengths and Difficulties questionnaire, parent version. Of 1101 eligible children, aged 5–7 years, 553 could be traced and analysed (274 in the LPV/r and 279 in the 3TC groups). Growth, clinical and neuropsychological outcomes did not differ between treatment groups. At school age, children exposed to LPV/r and 3TC at birth for 1 year had comparable growth and neuropsychological outcomes without evidence of long-term side-effects of LPV/r. It provides reassuring data on clinical outcomes for all HIV-infected children treated with this antiretroviral drug in early life.

## Introduction

The ANRS 12174 trial evaluated the use of LPV/r compared to lamivudine (3TC), as peri-exposure prophylaxis (PrEP) regimens to prevent postnatal mother-to-child HIV transmission during 12 months (i.e. the recommended duration of breastfeeding at the time of the trial) ^1^. It showed that these two regimens were equally effective to reduce postnatal transmission incidence below 1.5% in low-and-middle income countries (LMIC) ^2^. Both drugs were also equally well tolerated, with few drug-related adverse events. However, growth monitoring up to week 50 postnatal indicated that the LPV/r-based regimen was associated with reduced weight gain compared to the 3TC-based regimen ^3^. These findings were consistent with previous randomized trials reports indicating smaller weight-for-age z-scores in HIV-infected children treated with a LPV/r-based regimen compared to a nevirapine-based regimen ^4,5^. Although modest, the impact of LPV/r on growth may be associated with other co-morbidities such as a deleterious impact on neurodevelopment of the child. Indeed, restricted postnatal growth in the first years of life has been associated with reduced cognitive and motor development in childhood living in LMIC ^6,7^.

We therefore assessed whether this difference in growth persisted over time, at 5–7 years of age, and whether it was accompanied by differences in neurodevelopmental and clinical outcomes.

## Patients and methods

### Study design and population

The ANRS 12341 Mechanisms & Safety (M&S) study was a follow-up of children initially enrolled in the ANRS 12174, a multicentre randomized controlled trial conducted in four African countries, Burkina Faso, South Africa, Uganda and Zambia. The protocol and methodology of this trial have been published in detail elsewhere ^1,2^. Briefly, from November 2009 to May 2012, 1273 uninfected breastfed infants born to HIV-infected women who were not eligible to antiretroviral therapy (CD4 count > 350 cells per µL) as per WHO recommendations and national guidelines at the time, were randomly assigned (1:1) at day 7 after birth to receive either LPV/r or 3TC treatment as PrEP. Infants received pediatric formulations of either LPV/r (Kaletra, Abbott, Chicago, USA) or generic 3TC. Prophylaxis was given from day 7 until 1 week after cessation of breastfeeding or at the final visit of the trial at week 50. Pregnant women followed the routine national programs including for most of them an antiretroviral monotherapy from 28 weeks of amenorrhea until birth, then a single-dose nevirapine during labor, then a zidovudine/nevirapine prophylaxis until 7 day post-partum. Infants received nevirapine from birth until 7 days.

All ANRS 12174 trial participants who completed the 50-week follow-up free of HIV were eligible to this cross-sectional evaluation conducted between February 2017 and February 2018.

Mothers were contacted by study staff using the contact details collected during the trial. When necessary, home visits were conducted.

The mothers or caregivers and the children were invited to the study clinic for enrolment in a one- or two-day visit. The study was conducted in accordance with ethics principles contained in the World Medical Association (WMA) Declaration of Helsinki (Ethical Principles for Medical Research Involving Human Subjects) adopted by the 64th WMA General Assembly, Fortaleza, October 2013 and the ANRS Ethics charter for research in developing countries (May 2002, amended October 2008. Accessible at: http://www.anrs.fr/content/download/2807/16215/file/charte_ethiqueAngl2008.pdf). The protocol was approved by the relevant ethics committees, le Comité d’Ethique pour la Recherche en Santé (Burkina Faso), the Human Research Ethics Committee (South Africa), the Uganda National Council for Science and Technology (Uganda) and the Biomedical Research Ethics committee (Zambia).

### Clinical assessment

After informed consent given by the mother or the legal representative, anthropometric measurements were performed by trained staff using standardized material and procedures. The child’s weight, height and head circumference were measured twice to the nearest 0.1 kg (weight) or 0.1 cm (height and head circumference). The mean of the two nearest measurements was used in the analysis.

After medical history review with each mother/caregiver, the child underwent a clinical examination, including a detailed neurological examination. All procedures were standardized across the four countries, with common training. Socioeconomic questionnaires were also administered during the visit.

### Neuropsychological assessment

Children were assessed through a battery of questionnaires and tests designed to evaluate the core domains of mental status and cognitive performance. Tests were administered preferentially during mornings, by psychologists specifically trained together for this study. Tests and questionnaires were administrated in English or French or in the most common local languages in Zambia and Uganda. Translated versions (with back-translation) were available for the psychologists fluent in both the administrative and local languages. All incomplete tests were considered as invalid. Assessors were blinded for initial randomization groups.

The Strengths and Difficulties questionnaire (SDQ) was used to screen for mental health symptoms. The 25 items are summarized into 5 scales of 5 questions: (1) emotional symptoms, (2) conduct problem, (3) hyper activity, (4) peer relationship problems and, (5) prosocial behavior. The 4 first scales can be aggregated into a single global score reflecting the presence and severity of psychosocial difficulties. The questionnaire was administrated to the mother or caregiver.

The Kaufman Assessment Battery for children, second edition (KABC-II) ^8,9^, Luria model was used which measures cognitive abilities in three domains at the ages under study : (1) sequential processing, (2) simultaneous processing, (3) learning. A global score of mental processing index (MPI) was also obtained. The English and French versions of the manuals were used in the English and French speaking countries, respectively ^10,11^.

The Test of Variable of Attention (TOVA) is a computerized test assessing attention and impulsivity. TOVA measures the child’s response time, impulsivity, inattention and D-Prime (a measure of overall attention ability). We presented scores for impulsivity (commission error: responding inappropriately to the non target stimulus), as a proxy of inhibition, and computer auto-generated D prime (signal-detection sensitivity) as an overall measure of attention.

Finally, the Movement Assessment Battery for Children, second edition (MABC-2) identified impairments in motor performance. It allowed the assessment of: (1) manual dexterity, (2) aiming and catching and, (3) balance. Overall motor ability is assessed summing these 3 outcomes.

### Statistical analysis

Characteristics of eligible children enrolled versus non-enrolled in the M&S study were compared using Chi-square test or Fisher’s test as appropriate for categorical variables and Wilcoxon Mann–Whitney test for continuous variables. Factors associated with the probability of being enrolled were assessed using multivariable logistic regression. Participants’ characteristics were also presented and compared by treatment groups.

WHO Child Growth Standards were used to calculated length- or height-for-age z-score (LAZ or HAZ), weight-for-age z-score (WAZ), and weight-for-length z-score (WLZ) (for infants at 50 weeks) or body mass index (BMI) z-scores (for children of 5 years old and above). Stunting, underweight and wasting were defined as HAZ or LAZ < − 2, WAZ < − 2 and WLZ < − 2, respectively.

A socioeconomic score was computed by principal component analysis using 11 household assets and building materials for the house. This score was categorized in quintiles of increasing economic level.

Characteristics of children with three valid neuropsychological tests versus those with at least one invalid test were compared and factors associated with the probability of having all three neuropsychological tests valid were assessed using multivariable logistic regression. Scaled subtests and global scores of KABC-II, TOVA and MABC tests were used to assess association between neuropsychological function and treatment groups. For the SDQ questionnaire, as the scores were not normally distributed, they were dichotomized using standard UK cut-offs, in the absence of African references.

Mean differences between treatment groups (3TC-LPV/r) for the growth outcomes and for the 3 neuropsychological tests or odd-ratios (LPV/r vs 3TC) for SDQ25 and 95% confidence intervals were estimated using linear or logistic regressions.

Comparison of the different outcomes between treatment groups were adjusted for country, socio-economic level of the caregiver, number of children below 5 years of age in the household, education level and ability to read of the caregiver, gender and age of the child and, treatment duration, because these variables were considered as potential confounding factors. For example, the variable ‘number of children below 5 years in the household’ was included because its distribution differed substantially between treatment groups (Table 1) and could be associated with cognitive development as an indicator of socio-economic and education level (with higher number of young children being associated with poorer development). To account for losses to follow-up between the final visit of the trial and the M&S study, we adjusted estimates using an inverse probability weighting approach by modeling the participant’s probability of being enrolled in the M&S study ^12^. Treatment group and all variables with a *p* value < 0.20 for association with probability of being enrolled (see Supplementary Table 1) were entered in the model. The inverse of this probability was then used as weight applied to persons included in the multivariable regression analyses to correct for selection bias if any.

**Table 1:** Characteristics of ANRS12174 trial participants who subsequently enrolled in the M&S study by treatment group (N = 553).

	LPV/r (n = 274)	3TC (n = 279)	*P* value
**Mother’s characteristics at randomization (day 7)**			
Age (years)	28.2 (24.6;32.6)	27.8 (23.2;31.5)	0.12
Parity	3 (2;4)	2(1;4)	0.08
Pre-delivery CD4 counts (cells per uL)	520 (425;654)	517 (441;667)	0.26
Undetectable plasma HIV-1 RNA	123 (45.6)‡	132 (48.5)^¤¤^	0.49
WHO stage			0.83
1	264 (96.4%)	266 (95.3%)	
2	10 (3.6%)	12 (4.3%)	
3	0	1 (0.4%)	
Maternal PMTCT regimen			
During pregnancy	264 (96.3%)	265 (95.0%)	0.43
During labour	266 (97.1%)	273 (97.8%)	0.56
PMTCT treatment duration (weeks)	9 (6;12)	10 (6;12)	0.44
**Infant characteristics at randomization (day 7)**			
Sex			0.25
Male	130 (47.4%)	146 (52.3%)	
Female	144 (52.6%)	133 (47.7%)	
Birthweight (g)	3000 (2800;3400)	3100 (2800;3330)	0.87
**Infant characteristics at final (week 50) trial visit**			
Breastfeeding duration (weeks)	40.7 (33.2;44.9)†	41.0 (34.9;45.6)	0.20
Study treatment duration (weeks)	42.4 (33.4;47.4)	44.1 (36.9;48.9)	0.04
Weight (kg)	8.2 (7.5;9.1)	8.5 (7.7;9.2)^¤¤^	0.04
Lenght (cm)	71.4 (69.9;73.5)^+^	72.0 (70.2;73.5)^++^	0.09
WAZ	− 0.82 (− 1.70;0.01)^µ^	− 0.72 (− 1.59;0.30)^¤¤^	0.08
LAZ	− 1.10 (− 1.83;− 0.32)^+^	− 0.97 (− 1.64;− 0.23)^++^	0.25
WLZ	− 0.48 (− 1.30;0.46)^µ^	− 0.27 (− 1.13;0.51)^µµ^	0.15
Underweight (WAZ < − 2)	43 (19.1%)^¤^	37 (16.2%)^¤¤^	0.41
Stunting (HAZ < − 2)	49 (21.6%) ^+^	35 (15.0%)^++^	0.07
Wasting (WLZ < − 2)	23 (10.2%)^µ^	25 (11.0%)^µµ^	0.80
**Caregiver/household characteristics at M&S follow-up study**			
The mother has died	11 (4.0%)	7 (2.5%)	0.32
Mother’s BMI	24.7 (21.4;29.7)^£^	23.7 (20.9;29.4)^££^	0.21
The mother is taking HIV ART	163 (69.1%)^¥^	173 (68.7%)#	0.92
Caregiver’s education (highest grade)			
None	39 (14.2%)	27 (9.7%)	0.10
Any primary	102 (37.2%)	94 (33.7%)	
Any secondary	133 (48.6%)	158 (56.6%)	
The caregiver can read	221 (80.7%)	239 (85.7%)	0.12
The caregiver can write	219 (79.9%)	241 (86.4%)	0.04
Household socioeconomic score (quintiles)			
1 (poorest)	58 (21.2%)	53 (19.0%)	0.78
2	51 (18.6%)	59 (21.1%)	
3	51 (18.6%)	60 (21.5%)	
4	58 (21.2%)	53 (19.0%)	
5 (least poor)	56 (20.4%)	54 (19.3%)	
Number of adults living in household	2 (2;3)	2 (2;3)	0.71
Number of children (5–18 years) living in household	2 (2;3)	2 (1;3)	0.67
Number of children (< 5 years) living in household			
0	160 (58.4%)	130 (46.6%)	< 0.001
1	84 (30.7%)	128 (45.9%)	
≥ 2	30 (10.9%)	21 (7.5%)	
**Child’s characteristics at M&S follow-up study**			
Age (years)			
5	100 (36.5%)	111 (39.8%)	0.65
6	160 (58.4%)	152 (54.5%)	
7	14 (5.1%)	16 (5.7%)	
School attendance of the child			
None	53 (19.3%)	48 (17.2%)	0.20
Ever attended kindergarten or primary	147 (53.7%)	136 (48.8%)	
Attended both kindergarten and primary	74 (27.0%)	95 (34%)	

*LPV/r* lopinavir-ritonavir, *3TC* lamivudine, *PMTCT* prevention of mother to child transmission, *WAZ* weight-for-age Z-score, *LAZ* length-for-age Z-score, *WLZ* weight-for-length Z-score, *BMI* body mass index.

^†^2 Missing data. ^‡^4 Missing data. ^¤¤^7 Missing data. ^££^29 Missing data. ^#^27 Missing data. ^¥¥^30 Missing data. ^¥^38 Missing data. ^£^39 Missing data. ^++^46 Missing data. ^+^47 Missing data. ^¤^48 Missing data. ^µ^49 Missing data. ^¤¤^50 Missing data. ^µµ^52 Missing data.

A sensitivity analysis was performed in order to take into account KABC-II tests that were performed, but not completed and judged not valid by the testers, for reasons related to the behavior of the child such as unwillingness or bodily functions such as inability to do some of the physical tests. Missing scores for these tests were replaced by the lowest score recorded for complete and valid tests in the country site during the study and analyses were run again.

A multiple regression imputation (100 imputations) of data for the tests (KABC-II, TOVA or MABC-2) that were not performed, was done using chained equation imputation and assuming that the data was missing at random. Linear regression analysis was done on the imputed dataset.

Three summary clinical outcomes were defined as follows: proportion of children with at least one abnormality observed by the physician; proportion of children who had, during the previous year, at least one consultation in a clinic or outpatient hospital without being admitted, and proportion of children ever admitted to hospital since the ANRS 12174 week 50 visit. These outcomes were compared by treatment group using a chi-square test. Odds-ratios (LPV/r vs 3TC) for these clinical outcomes and 95% confidence intervals were also estimated using multivariable logistic regressions with inverse probability weighting.

We estimated the mortality rate, from inclusion in ANRS 12174 (day 7) to M&S study, and from ANRS 12174 week 50-visit to M&S study, using the Kaplan–Meier procedure and their 95% confidence interval after log–log transformation. Cumulative probability of mortality was compared between treatment groups using the log rank test.

## Results

**Figure 1:**
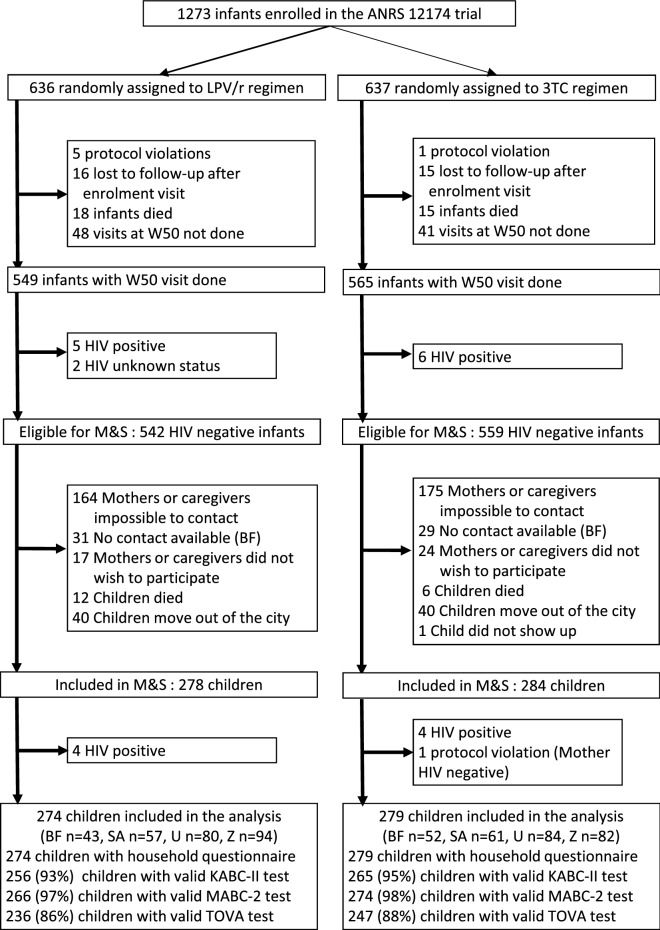
Study profile. *BF* Burkina Faso, *SA* South Africa, *U* Uganda, *Z* Zambia.

Of 1273 infants enrolled in the trial, 1101 were eligible for enrollment in the M&S study after excluding infants who did not complete the 50 week visit (n = 159) and those who were either HIV-positive (n = 11) or had an unknown HIV status at week 50 (n = 2) (Fig. 1). A total of 562 HIV-exposed and uninfected children were enrolled between February 2017 and February 2018. The proportion of children enrolled was lower in Burkina Faso (50%) and Zambia (38%) than in Uganda (68%) and South Africa (62%), but proportions were similar between treatment groups (LPV/r or 3TC) (Supplementary Table 1). Enrolled children had older (28.1 (± 5.6) vs 27.0 (± 5.3) years) and more educated mothers (53.0% vs 46.7% with secondary level or more) and were breastfed for a shorter duration (37.9 (± 11.0) vs 38.9 (± 11.0) weeks) than their non-enrolled counterparts. Country, age and education level of the mother remained independently associated with probability of being enrolled in the follow-up study in a multivariable analysis. A full description of enrolled and non-enrolled eligible children by site and treatment group is available in supplementary tables (Supplementary Tables 2, 3, 4, 5).

### Baseline and follow-up characteristics of M&S participants

A total of 553 children were included in the analysis after exclusion of 9 children (8 HIV-positive children at enrollment in the M&S study and 1 protocol deviation) (Fig. 1) ^13^. Most baseline and M&S characteristics of children and their mothers included in the M&S follow-up study were well balanced between treatment groups (Table 1). Children in the LPV/r group had lower weight at week 50, and received study prophylaxis for a shorter duration. In addition, at follow-up, mothers or caregivers of the participants in the LPV/r group were more likely to live in household without young children (< 5 years of age).

The children’s age during the M&S study ranged from 5 to 7 years. Only 30 children were 7 years old. Overall, 69% of mothers reported taking antiretroviral treatment.

### Growth outcomes

**Figure 2:**
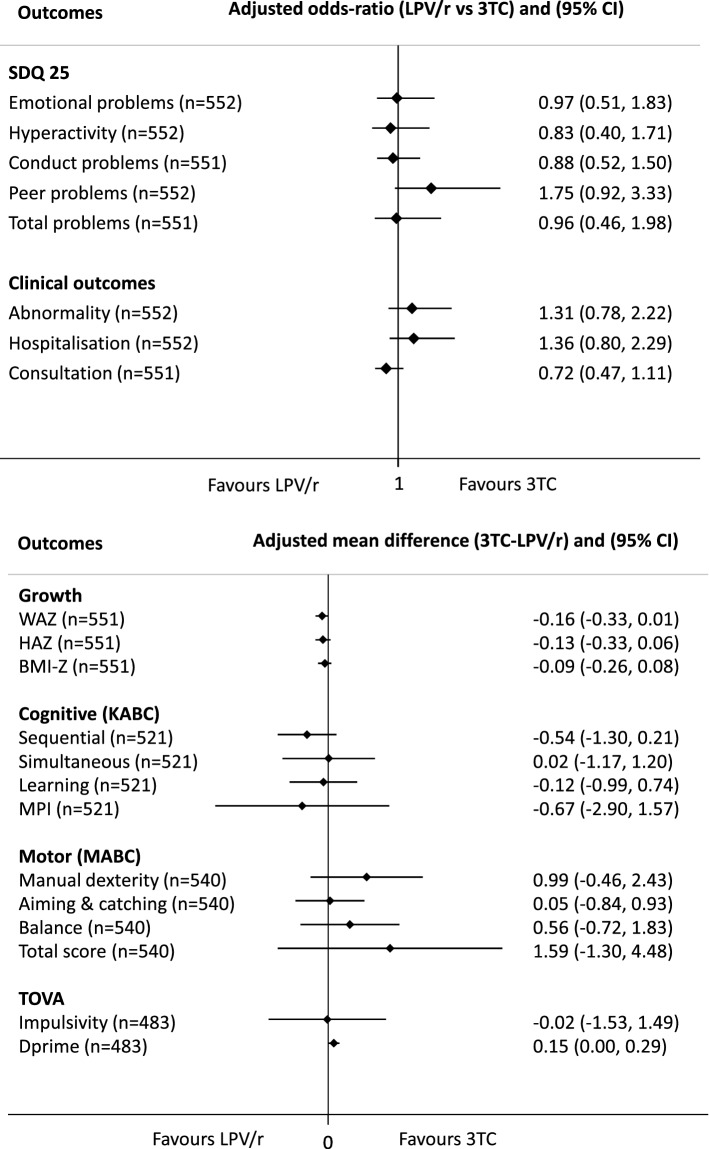
Comparison of growth, neuro-psychological and clinical outcomes by treatment groups. Note: Estimates were adjusted for country, socio-economic level of the caregiver, number of children below 5 years of age in the household, education level and ability to read of the caregiver, gender and age of the child and treatment duration. Inverse probability weighting was applied to account for selection bias. *LPV/r* lopinavir-ritonavir, *3TC* lamivudine, *CI* confidence interval, *SDQ* strengths and difficulties questionnaire, *WAZ* weight-for-age z-score, *LAZ* length-for-age z-score, *BMI-z* body mass index z-score, *KABC* Kaufman Assessment Battery for children, *MPI* mental processing index, *MABC* Movement Assessment Battery for Children, *TOVA* test of variables of attention. DPrime is an overall measure of attention.

At the final visit of the trial (week 50), children included in the analysis tended to be shorter, lighter and thinner in the LPV/r group than in the 3TC group, although differences were not statistically different (Table 1). At the M&S visit, z-scores were similar in both treatment groups in crude or adjusted analyses (Table 2 and Fig. 2).

**Table 2:** Clinical outcomes of participants at M&S follow-up study by treatment group (N = 553).

	LPV/r (n = 274)	3TC (n = 279)	*P* value
**Anthropometry**			
Weight (kg)	19.3 (17.6;20.8)	19.2 (17.4;20.6)	0.31
Height (cm)	113.5 (110.0;117.0)	113.2 (109.0;117.0)	0.55
Head circumference (cm)	51.5 (50.6;52.0)	51.5 (50.5;52.2)	0.91
WAZ	− 0.50 (− 1.07;− 0.01)†	− 0.55 (− 1.20;− 0.03)†	0.24
HAZ	− 0.52 (− 1.19;0.01)†	− 0.55 (− 1.31;0.06)†	0.65
BMI z-score	− 0.27 (− 0.87;0.31)†	− 0.41 (− 0.96;0.32)†	0.17
Underweight (WAZ < − 2)	12 (4.4%)†	18 (6.5%)†	0.28
Stunting (HAZ < − 2)	14 (5.1%)†	23 (8.3%)†	0.14
**Clinical examination**			
At least one abnormality observed	94 (34.6%)†	87 (31.2%)	0.40
Type of event			
Neurological system	3	1	
Cardiac system	11	2	
Respiratory system	16	19	
Dental system	48	51	
Abdomen and genital urinary	18	19	
Sensory system	29	20	
**Medical history**			
Any medical consultation in clinic or outpatient clinic (without being admitted) during the last year	178 (65.2%)‡	190 (68.1%)	0.22
Type of event§			
Neurological (convulsions)	6	4	
Cardiac	0	0	
Gastrointestinal	27	30	
Respiratory	80	89	
Skin	25	16	
Malaria	76	82	
Malnutrition	0	0	
Other	31	44	
Unknown	10	4	
Ever been admitted to hospital since the last trial visit	58 (21.3%)†	60 (21.5%)	0.95
Type of event§			
Neurological (convulsions)	6	22	
Cardiac	0	0	
Gastric	18	22	
Respiratory	19	20	
Skin	0	0	
Malaria	46	71	
Cerebral malaria	14	7	
Malnutrition	4	5	
Other	18	13	
Unknown	2	4	

^†^1 Missing data ^‡^2 Missing data ^§^At least one consultation or hospital admission for this type of event.

*LPV/r* lopinavir-ritonavir, *3TC* lamivudine, *WAZ* weight-for-age z-score, *HAZ* height-for-age z-score, *BMI* body mass index.

### Neurodevelopmental outcomes

Of the 553 children included in the analysis, 463 had all three valid neuropsychological tests completed. 521 (94.2%), 540 (97.6%) and 483 (87.3%) children had a valid test for KABC-II, MABC-2 and TOVA, respectively. The children with completed tests had more educated mothers and were breastfed for a longer duration (Supplemental Table 6) than those without all tests. The proportion of completed valid tests did not differ by treatment groups.

There were no differences found on the SDQ, KABC-II or MABC-2 scales between treatment groups in adjusted analyses (Fig. 2). Scores were similar for all subtests as well as for combined or total scoring scales across both trial groups. However, for the TOVA overall attention score (Dprime), a difference was observed between groups with a tendency for better performance in the 3TC group. This difference did not remain significant after applying the Benjamini–Hochberg adjustment to control for the false discovery rate in the analysis of multiple outcomes ^14^.

In the sensitivity analyses, missing scores in 14 children with invalid KABC-II test were replaced by the lowest score of their study site and results of the analysis were unchanged (data not shown). Performance in KABC-II, MABC-2 and TOVA were also compared by treatment groups with multiple imputation of missing scores for tests that were not performed (18, 21 and 9 tests not performed for KABC-II, MABC-2 and TOVA, respectively) and no difference was found (data not shown).

### Mortality and morbidity

Of the 1273 HIV-1 exposed infants at the trial enrolment, six were wrongly enrolled and 31 never came back after enrollment, leaving 1236 infants included in the mortality assessment. Of these, 399 were lost to follow-up without information regarding their vital status when the M&S follow-up study commenced. Overall 51 deaths were recorded; 33 infants died between day 7 and week 50 (duration of the trial), and 18 additional deaths were reported at follow-up. The cumulative mortality rate from birth to follow-up was 7.93% (95% CI 4.95–12.59) in children assigned to LPV/r regimen and 4.16% (95% CI 2.69–6.39) in those assigned to 3TC regimen (p-value for log-rank test = 0.18). Mortality rate from week 50 to follow-up was 5.05% (95% CI 2.47; 10.17) in LPV/r group and 1.69% (95% CI 0.76; 3.72) in the 3TC group; with p-value for log-rank test = 0.14.

From week 50 to the start of the M&S study inclusion, proportions of reported admission to hospital were similar between treatment groups (58/274 (21.3%) in the LPV/r group versus 60/279 (21.5%) in the 3TC group) as well as proportions of children with at least one consultation to clinic or outpatient hospital. Proportions of children with at least one abnormality observed during clinical examination of the M&S visit were also similar in the two treatment groups (Table 2 and Fig. 2).

## Discussion

This study assessed the long-term safety outcomes in 553 HIV-exposed uninfected children who received either LPV/r or 3TC prophylactic regimens during breastfeeding for maximum 50 weeks to prevent HIV. We have previously reported that children who received LPV/r gained less weight at week 50 than those who received 3TC ^3^. Here, we found that this difference in weight gain did not persist over time after drug discontinuation. At school age (5–7 years) weight-for-age, height-for-age and BMI z-scores were similar in the two groups.

HIV-infected children treated with LPV/r have also experienced persistent slower growth than their counterparts receiving NNRTI-based regimen (4,5). Switching children to a NNRTI-based treatment after initial suppression with a LPV/r-based treatment has been associated with slightly better growth outcomes ^15^. Altogether these findings provide consistent support for an adverse impact of LPV/r on early infant growth that is reversible after drug discontinuation. This adverse impact needs to be balanced by the benefit of this drug on viral suppression in comparison with NNRTI-based regimen.

A large number of studies have compared neurodevelopmental outcomes between HIV-infected, HIV-exposed but uninfected and HIV-unexposed children ^16–21^.While sub-optimal cognitive performance have been reported in HIV-infected children, with partial improvement when ART is started early, the studies assessing the role of HIV exposure provided discrepant conclusions: some reported reduced cognitive performance in exposed children, while others did not find any differences after taking into account socio-economic and cultural confounding factors ^16–18,20,22^.

In this study, we hypothesized that the impact of LPV/r on early postnatal growth could be associated with adverse effects on neurodevelopment with long term effects, based on a large empirical literature on the relationship between linear growth and cognitive outcomes among school age children ^6^. This hypothesis was not supported by our findings. Indeed, crude or adjusted differences in scores of the different tests and subtests were not statistically significant at a 5% level.

We are not aware of any other study evaluating the impact of LPV/r on child neurodevelopment when the drug is directly given to uninfected children as prophylaxis, but there are several studies in which uninfected children were exposed in utero or through breastfeeding to LPV/r given to mothers as part of their treatment.

Consistently with our findings, neurocognitive outcomes of uninfected children, born to HIV-infected mothers who received either a triple NRTI or a PI-based regimen (LPV/r-zidovudine) in the Mma Bana trial, assessed at 24 months of age were similar between treatment groups ^23,24^. In contrast, in-utero exposure to PI-based compared to NNRTI-based regimens was associated with higher cognitive performance in an observational longitudinal study among HIV-exposed children ^25^. In a recent report, Boivin and colleagues compared neurodevelopmental outcomes at 12, 24, 48 and 60 months between HIV-exposed uninfected children, who were exposed to various combinations of ante-partum and post-partum maternal ARVs, and HIV-unexposed children from Uganda and Malawi ^22^. Exposure to maternal LPV/r-based triple ARV regimen in-utero was not associated with lower cognitive performance when compared to absence of exposure (in HIV-unexposed uninfected children) and was associated with slightly higher Mullen Scales of Early Learning scores when compared to maternal zidovudine prophylaxis exposure. The authors hypothesized that maternal triple ARV might confer protection of the mother’s health with benefits for the child’s development.

The strengths of this study include the multi-site randomized design; with assessors blinded to treatment group, the use of standardized and validated tests which were administered by trained staff and the large sample size. The ANRS 12174 design, where ARVs are given as prophylaxis to uninfected infants early in life, also allowed us to compare neurodevelopmental safety of two drugs without interference of either HIV infection or other drugs used in combination. This study has limitations. The participation rate in the follow-up study was low and the causes of this low rate were diverse and specific to each country, but evenly balanced between treatment groups. Children enrolled in the follow-up study differed slightly from those who were not enrolled and these differences could limit the generalizability of the results to the original ANRS 12174 trial population. However, the use of inverse probability weighting allowed us to correct for selection bias and estimate the treatment effect in a pseudo-population similar to the original eligible population.

At the time of the study, the respective tests used were not normed in the four study countries, thus the test-associated European or US norms were used to scale the raw score of neuropsychological tests according to the age of the child. Therefore, one need to interpret these scaled scores with care. The use of a local norm should be preferred for purposes beyond comparisons of exposures ^26^. As our main objective was to compare outcomes between the two treatment groups we believe the use of these tests are justifiable. In the absence of comparison to a control group of children who are HIV unexposed and uninfected, we cannot exclude that both treatments have adverse effects on neuropsychological development. Indeed, 3TC is a NRTI, a class of antiretrovirals that induce mitochondrial genotoxicity with potential adverse impact on neurodevelopment of the child when LPV/r could act through oxidative stress on mitochondrial toxicity ^27,28^.

## Conclusion

The impact at 1 year of LPV/r prophylaxis on growth did not persist over time after drug withdrawal. At school age, children exposed to LPV/r or 3TC regimens during their first year of life had comparable growth and neuropsychological outcomes without evidence of long-term deleterious effects of LPV/r on health status. This study provides reassuring data regarding the use of this antiretroviral drug in early life.

## Supplementary Information

Supplementary Information 1.
