# Supplementary material for: Growth, clinical and neurodevelopmental outcomes at school age are similar for children who received 1-year lamivudine or lopinavir/ritonavir HIV prophylaxis in early life
Source: Sci Rep. 2021 Feb 4;11:3173. doi: 10.1038/s41598-021-82762-8 (PMC7862474; doi:10.1038/s41598-021-82762-8)
Supplement: Supplementary file 1 — Supplementary Information 1. [file 41598_2021_82762_MOESM1_ESM.docx]

**Supplementary information**

**Growth, clinical and neurodevelopmental outcomes at school age are similar for children who received 1-year lamivudine or lopinavir/ritonavir HIV prophylaxis in early life**

Nicolas Nagot^1^; Mandisa Singata-Madliki^2^; Amandine Cournil^1^; Joyce Nalugya^3^; Souleymane Tassembedo^4^; Catherine Quillet^1^; Melany W. Tonga^5^; James Tumwine^3^; Nicolas Meda^4^; Chipepo Kankasa^5^; Mwiya Mwiya^5^; Paul Bangirana^3^; Marianne Peries^1^; Joanne Batting ^2^, Ingunn M.S. Engebretsen^6^; Thorkild Tylleskär^6^; Philippe Vande Perre^1^; Grace Ndeezi^3^; Jean-Pierre Molès^1^

1- Pathogenesis and control of chronic infections, INSERM, Etablissement Français du Sang, University of Montpellier, Montpellier, France

2- University of Fort Hare, East London, South Africa

3- School of Medicine, College of Health Sciences, Makerere University, Kampala, Uganda

4- Centre MURAZ, Bobo-Dioulasso, Burkina Faso

5- Department of Paediatrics and Child Health, University Teaching Hospital, Lusaka, Zambia

6- Centre for International Health, University of Bergen, Bergen, Norway

Supplementary Table 1. Characteristics of trial participants who did and did not subsequently enroll into the M&S study and multivariable analysis of predictors of enrolment

|  |  | | | Multivariable analysis^b^ | | |  |
| --- | --- | --- | --- | --- | --- | --- | --- |
|  | **Not enrolled (n=539)** | **Enrolled (n=562)** | P value^a^ | | aOR 95%CI | P value | |
| **Randomization group** |  |  |  | |  |  | |
| LPV/r | 264 (49.0) | 278 (49.5) | 0.87 | |  |  | |
| 3TC | 275 (51.0) | 284 (50.5) |  | |  |  | |
| **Country site** |  |  |  | |  |  | |
| Burkina Faso | 99 (18.4) | 98 (17.4) | <0.001 | | 1 |  | |
| South Africa | 72 (13.4) | 120 (21.4) |  | | 1.19 (0.69;2.06) | 0.53 | |
| Uganda | 78 (14.5) | 166 (29.5) |  | | 1.98 (1.22;3.22) | 0.006 | |
| Zambia | 290 (53.8) | 178 (31.7) |  | | 0.55 (0.36;0.84) | 0.006 | |
| **Mother** |  |  |  | |  |  | |
| Age (years) | 26.1 (22.9;30.4) | 28.1 (24.0;32.0) | <0.001 | | 1.05 (1.03;1.08) | <0.001 | |
| Parity | 2.7 (± 1.45) | 2.9 (± 1.66) | 0.05 | |  |  | |
| Pre-delivery CD4 counts (cells per µL) | 542 (432;678) | 519 (432;658) | 0.17 | |  |  | |
| Plasma HIV-1 RNA |  |  |  | |  |  | |
| Undetectable | 226 (42.9) | 258 (46.8) | 0.19 | |  |  | |
| Median (log10 copies/mL) | 3.45 (2.95;3.92) | 3.39 (2.93;3.90) | 0.27 | |  |  | |
| WHO clinical HIV-1 stage |  |  |  | |  |  | |
| 1 | 523 (97.2) | 539 (95.9) | 0.32 | |  |  | |
| 2 | 15 (2.8) | 22 (3.9) |  | |  |  | |
| 3 | 0 | 1 (0.2) |  | |  |  | |
| PMTCT regimen |  |  |  | |  |  | |
| During pregnancy | 519 (96.3) | 538 (95.7) | 0.64 | |  |  | |
| During labour | 529 (98.1) | 547 (97.3) | 0.36 | |  |  | |
| Highest education level completed |  |  |  | |  |  | |
| None | 90 (16.7) | 62 (11.0) | 0.01 | | 1 |  | |
| Primary | 197 (36.5) | 202 (35.9) |  | | 1.39 (0.86;2.25) | 0.18 | |
| Secondary or tertiary | 252 (46.7) | 298 (53.0) |  | | 1.87 (1.15;3.03) | 0.01 | |
| **Infant** |  |  |  | |  |  | |
| Sex |  |  |  | |  |  | |
| Boys | 289 (53.6) | 279 (49.6) | 0.21 | |  |  | |
| Girls | 250 (46.4) | 283 (50.4) |  | |  |  | |
| Birthweight (g) | 3,000 (2,750;3,350) | 3,020 (2,800;3,400) | 0.12 | |  |  | |
| Breastfeeding duration (weeks) | 41.6 (35.4;46.7) | 40.8 (34.5;45.3) | 0.04 | |  |  | |
| Mean WAZ (SD) | -1.03 (1.22) | -0.77 (1.29) | <0.001 | |  |  | |
| Mean LAZ (SD) | -1.18 (1.07) | -1.01 (1.09) | 0.014 | |  |  | |
| Mean WLZ (SD) | -0.59 (1.23) | -0.37 (1.31) | 0.002 | |  |  | |

Values are median (interquartile range) for continuous variable and no. (percentage) for categorical variable, unless otherwise specified.

^a^P value for chi-square or Wilcoxon Mann-Whitney tests.

^b^Variables associated with the outcomes in bivariable regression analysis with a p<0.25 were included in a multivariable regression model. A backward procedure was used to select the final model.

Supplementary Table 2. Characteristics of trial participants who did and did not subsequently enroll into the M&S study in Burkina Faso by treatment group

| **Burkina Faso** | **LPV/r (n=97)** | | | **3TC (n=100)** | | |
| --- | --- | --- | --- | --- | --- | --- |
| **variable** | **Not included (n=53)** | **Included (n=44)** | **P** | **Not included (n=46)** | **Included (n=54)** | **P** |
| **Mother** |  |  |  |  |  |  |
| Age (years) | 26.97 (23.75;31.15) | 30.07 (26.44;34.06) | 0.02 | 28.50 (25.02;32.24) | 28.18 (23.02;31.42) | 0.35 |
| Parity | 2.72 (± 1.57) | 3.20 (± 1.47) | 0.05 | 2.98 (± 1.73) | 2.52 (± 1.68) | 0.10 |
| Pre-delivery CD4 counts (cells per µL) | 558 (433;681) | 536 (419;639) | 0.39 | 480 (395;607) | 519 (447;687) | 0.03 |
| Plasma HIV-1 RNA at enrolment  Undetectable | 25 (47) | 15 (34) | 0.19 | 22 (47) | 27 (50) | 0.83 |
| Median (log_10_ copies per mL) | 3.29 (2.96;3.72) | 3.25 (3.04;3.74) | 0.65 | 3.20 (2.73;3.51) | 3.13 (2.74;3.69) | 0.68 |
| WHO clinical HIV-1 stage  1 | 51 (97) | 40 (91) | 0.41 | 38 (84) | 53 (98) | 0.02 |
| 2 | 2 (3) | 4 (9) |  | 7 (16) | 1 (2) |  |
| PMTCT regimen  During pregnancy | 52 (98) | 43 (97) | 1.00 | 45 (97) | 53 (98) | 1.00 |
| During labour | 46 (86) | 38 (86) | 0.95 | 43 (93) | 48 (88) | 0.50 |
| Highest education level completed  None | 24 (45) | 26 (59) | 0.16 | 28 (61) | 18 (33) | 0.01 |
| Primary | 15 (28) | 13 (30) |  | 11 (24) | 14 (26) |  |
| Secondary or tertiary | 14 (27) | 5 (11) |  | 7 (15) | 22 (41) |  |
| **Infant** |  |  |  |  |  |  |
| Gender  Boys | 33 (63) | 21 (47) | 0.15 | 33 (72) | 26 (48) | 0.02 |
| Girls | 20 (37) | 23 (53) |  | 13 (28) | 28 (52) |  |
| Birthweight (g) | 2,940 (2,700;3,300) | 2,890 (2,700;3,160) | 0.63 | 2,775 (2,500;3,150) | 3,000 (2,750;3,300) | 0.07 |
| Breastfeeding duration (weeks) | 46.57 (45.29;48.43) | 46.07 (44.21;47.71) | 0.26 | 47.57 (45.43;49.57) | 46.29 (45.14;48.71) | 0.16 |
| WHZ 50 weeks | -1.39 (-1.84;-0.28) | -1.09 (-1.78;-0.84) | 0.62 | -1.05 (-1.83;-0.51) | -0.70 (-1.43;-0.14) | 0.15 |
| HAZ 50 weeks | -0.83 (-1.91;-0.52) | -0.88 (-1.82;-0.09) | 0.66 | -0.89 (-1.43;-0.10) | -0.80 (-1.16;0.16) | 0.37 |
| WAZ 50 weeks | -1.58 (-2.10;-0.56) | -1.26 (-2.11;-0.63) | 0.89 | -1.16 (-1.97;-0.63) | -0.79 (-1.69;-0.06) | 0.13 |

Supplementary Table 3. Characteristics of trial participants who did and did not subsequently enroll into the M&S study in South Africa by treatment group

| **South Africa** | **LPV/r (n=92)** | | | **3TC (n=100)** | | |
| --- | --- | --- | --- | --- | --- | --- |
| **variable** | **Not included (n=34)** | **Included (n=58)** | **P** | **Not included (n=38)** | **Included (n=62)** | **P** |
| **Mother** |  |  |  |  |  |  |
| Age (years) | 26.25 (23.71;34.66) | 29.31 (25.38;34.42) | 0.57 | 27.26 (22.93;30.46) | 26.73 (22.58;32.05) | 0.83 |
| Parity | 2.03 (± 0.83) | 2.21 (± 1.06) | 0.55 | 2.00 (± 0.99) | 1.97 (± 1.07) | 0.78 |
| Pre-delivery CD4 counts (cells per µL) | 505 (432;679) | 490 (421;630) | 0.52 | 537 (426;678) | 495 (430;654) | 0.81 |
| Plasma HIV-1 RNA at enrolment  Undetectable | 24 (70) | 25 (43) | 0.01 | 19 (50) | 33 (53) | 0.75 |
| Median (log_10_ copies per mL) | 3.24 (2.93;3.61) | 3.46 (2.76;4.02) | 0.46 | 3.36 (2.86;3.76) | 3.13 (2.77;3.61) | 0.22 |
| WHO clinical HIV-1 stage  1 | 34 (100) | 57 (98) | 1.00 | 38 (100) | 60 (97) | 0.52 |
| 2 | 0 (0) | 1 (2) |  | 0 (0) | 2 (3) |  |
| PMTCT regimen  During pregnancy | 34 (100) | 58 (100) |  | 38 (100) | 61 (98) | 1.00 |
| During labour | 34 (100) | 56 (97) | 0.53 | 38 (100) | 62 (100) |  |
| Highest education level completed  None | 0 (0) | 1 (2) | 1.00 | 0 (0) | 1 (2) | 0.68 |
| Primary | 3 (9) | 6 (10) |  | 4 (11) | 4 (6) |  |
| Secondary or tertiary | 31 (91) | 51 (88) |  | 34 (89) | 57 (92) |  |
| **Infant** |  |  |  |  |  |  |
| Gender  Boys | 17 (50) | 30 (52) | 0.87 | 16 (42) | 36 (58) | 0.12 |
| Girls | 17 (50) | 28 (48) |  | 22 (58) | 26 (42) |  |
| Birthweight (g) | 3,150(2,790;3,480) | 3,095(2,860;3,480) | 0.78 | 3,185 (2,820;3,400) | 3,250 (2,750;3,500) | 0.92 |
| Breastfeeding duration (weeks) | 23.79 (9.07;44.07) | 28.29 (10.71;46.14) | 0.50 | 39.50 (16.43;47.00) | 35.71 (25.00;48.57) | 0.60 |
| WHZ 50 weeks | 0.96 (0.29;1.78) | 0.50 (0.20;1.10) | 0.43 | 0.79 (-0.49;1.92) | 0.92 (-0.18;1.44) | 0.63 |
| HAZ 50 weeks | -0.70 (-1.80;0.35) | -0.73 (-1.39;-0.18) | 0.85 | -0.73 (-1.35;-0.04) | -0.57 (-1.15;0.15) | 0.65 |
| WAZ 50 weeks | 0.33 (-0.54;1.16) | 0.13 (-0.41;0.67) | 0.83 | 0.07 (-0.55;1.30) | 0.45 (-0.61;1.46) | 0.52 |

Supplementary Table 4. Characteristics of trial participants who did and did not subsequently enroll into the M&S study in Uganda by treatment group

| **Uganda** | **LPV/r (n=125)** | | | **3TC (n=119)** | | |
| --- | --- | --- | --- | --- | --- | --- |
| **variable** | **Not included (n=43)** | **Included (n=82)** | **P** | **Not included (n=35)** | **Included (n=84)** | **P** |
| **Mother** |  |  |  |  |  |  |
| Age (years) | 25.22 (23.02;28.10) | 27.10 (23.12;30.07) | 0.22 | 24.81 (22.13;28.08) | 28.09 (23.13;30.19) | 0.05 |
| Parity | 3.56 (± 1.84) | 3.62 (± 2.06) | 0.93 | 3.00 (± 1.81) | 3.69 (± 2.10) | 0.11 |
| Pre-delivery CD4 counts (cells per µL) | 539 (436;704) | 522 (429;615) | 0.41 | 550 (479;648) | 527 (441;612) | 0.33 |
| Plasma HIV-1 RNA at enrolment  Undetectable | 24 (56) | 60 (73) | 0.05 | 24 (69) | 50 (63) | 0.59 |
| Median (log_10_ copies per mL) | 3.63 (2.93;3.76) | 3.67 (2.85;3.99) | 0.70 | 3.63 (3.00;3.87) | 3.57 (3.08;5.21) | 0.71 |
| WHO clinical HIV-1 stage  1 | 39 (91) | 77 (94) | 0.49 | 33 (94) | 75 (89) | 0.80 |
| 2 | 4 (9) | 5 (6) |  | 2 (6) | 8 (10) |  |
| 3 | 0 | 0 |  | 0 (0) | 1 (1) |  |
| PMTCT regimen  During pregnancy | 32 (74) | 73 (89) | 0.03 | 28 (80) | 72 (86) | 0.44 |
| During labour | 43 (100) | 82 (100) |  | 35 (100) | 83 (99) | 1.00 |
| Highest education level completed  None | 3 (7) | 4 (5) | 0.60 | 2 (6) | 3 (4) | 0.08 |
| Primary | 29 (67) | 50 (61) |  | 26 (74) | 47 (56) |  |
| Secondary or tertiary | 11 (26) | 28 (34) |  | 7 (20) | 34 (40) |  |
| **Infant** |  |  |  |  |  |  |
| Gender  Boys | 17 (40) | 41 (50) | 0.27 | 16 (46) | 43 (51) | 0.59 |
| Girls | 26 (60) | 41 (50) |  | 19 (54) | 41 (9) |  |
| Birthweight (g) | 3,000(2,700;3,300) | 3,000(2,700;3,300) | 0.31 | 3,100 (2,800;3,500) | 3,100 (2,930;3,350) | 0.59 |
| Breastfeeding duration (weeks) | 40.79 (34.29;43.00) | 40.43 (36.57;43.00) | 0.98 | 39.43 (34.43;43.71) | 40.43 (36.93;43.21) | 0.54 |
| WHZ 50 weeks | -0.45 (-1.62;0.42) | -0.44 (-0.95;0.46) | 0.67 | -0.14 (-0.93;0.56) | -0.05 (-0.96;0.47) | 0.86 |
| HAZ 50 weeks | -1.42 (-2.01;-0.70) | -1.40 (-2.12;-0.35) | 0.99 | -1.02 (-1.49;-0.28) | -0.82 (-1.61;-0.10) | 0.96 |
| WAZ 50 weeks | -0.52 (-2.14;-0.13) | -1.02 (-1.70;0.14) | 0.93 | -0.71 (-1.36;0.14) | -0.61 (-1.30;0.30) | 0.95 |

Supplementary Table 5. Characteristics of trial participants who did and did not subsequently enroll into the M&S study in Zambia by treatment group

| **Zambia** | **LPV/r (n=228)** | | | **3TC (n=240)** | | |
| --- | --- | --- | --- | --- | --- | --- |
| **variable** | **Not included (n=134)** | **Included (n=94)** | **P** | **Not included (n=156)** | **Included (n=84)** | **P** |
| **Mother** |  |  |  |  |  |  |
| Age (years) | 26.24 (22.44;30.40) | 27.62 (24.59;33.05) | 0.01 | 25.73 (22.57;30.06) | 27.66 (24.57;32.20) | 0.01 |
| Parity | 2.67 (± 1.31) | 2.82 (± 1.19) | 0.22 | 2.53 (± 1.26) | 2.74 (± 1.28) | 0.20 |
| Pre-delivery CD4 counts (cells per µL) | 544 (424;669) | 491 (414;714) | 0.60 | 572 (450;712) | 541 (446;702) | 0.66 |
| Plasma HIV-1 RNA at enrolment  Undetectable | 39 (30) | 25 (27) | 0.72 | 49 (33) | 23 (28) | 0.43 |
| Median (log_10_ copies per mL) | 3.57 (2.93;4.25) | 3.37 (3.01;3.97) | 0.16 | 3.49 (3.06;4.35) | 3.47 (3.19;3.85) | 0.60 |
| WHO clinical HIV-1 stage  1 | 134 (100) | 94 (100) |  | 156 (100) | 83 (99) | 0.35 |
| 2 |  |  |  | 0 (0) | 1 (1) |  |
| PMTCT regimen  During pregnancy | 134 (100) | 94 (100) |  | 156 (100) | 84 (100) |  |
| During labour | 134 (100) | 94 (100) |  | 156 (100) | 84 (100) |  |
| Highest education level completed  None | 13 (10) | 2 (2) | 0.07 | 20 (13) | 7 (8) | 0.56 |
| Primary | 54 (40) | 38 (40) |  | 55 (35) | 30 (36) |  |
| Secondary or tertiary | 67 (50) | 54 (58) |  | 81 (52) | 47 (56) |  |
| **Infant** |  |  |  |  |  |  |
| Gender  Boys | 72 (54) | 40 (42) | 0.10 | 85 (54) | 43 (51) | 0.63 |
| Girls | 62 (46) | 54 (58) |  | 71 (46) | 41 (49) |  |
| Birthweight (g) | 3,000(2,800;3,300) | 3,100 (2,800;3,480) | 0.07 | 3,000 (2,800;3,400) | 3,000 (2,760;3,240) | 0.57 |
| Breastfeeding duration (weeks) | 40.00 (34.43;44.71) | 38.86 (32.43;42.71) | 0.09 | 40.86 (36.00;45.14) | 38.43 (35.00;42.71) | 0.08 |
| WHZ 50 weeks | -0.76 (-1.39;-0.03) | -0.66 (-1.44;0.30) | 0.50 | -0.80 (-1.47;-0.10) | -0.61 (-1.40;0.20) | 0.23 |
| HAZ 50 weeks | -1.46 (-1.88;-0.71) | -1.23 (-1.80;-0.52) | 0.25 | -1.42 (-2.10;-0.73) | -1.48 (-1.99;-0.61) | 0.96 |
| WAZ 50 weeks | -1.28 (-1.94;-0.59) | -1.04 (-1.84;-0.13) | 0.24 | -1.35 (-2.00;-0.54) | -1.25 (-2.06;-0.18) | 0.21 |

Supplementary Table 6. Characteristics of participants with at least one invalid test and with all three neuropsychological tests valid and multivariable analysis of factors associated with tests validity

|  |  | | | Multivariable analysis^b^ | | |  |
| --- | --- | --- | --- | --- | --- | --- | --- |
|  | **At least one invalid test (n=90)** | **All three tests valid (n=463)** | P^a^ | | aOR 95%CI | P | |
| **Randomization group** |  |  |  | |  |  | |
| LPV/r | 50 (18.2) | 224 (81.7) | 0.21 | |  |  | |
| 3TC | 40 (14.3) | 239 (85.7) |  | |  |  | |
| **Country site** |  |  |  | |  |  | |
| Burkina Faso | 18 (18.9) | 77 (81.0) | <0.001 | | Ref. |  | |
| South Africa | 43 (36.4) | 75 (63.6) |  | | 0.27 (0.11;0.66) | 0.004 | |
| Uganda | 6 (3.7) | 158 (96.3) |  | | 6.62 (2.16; 20.27) | 0.001 | |
| Zambia | 23 (13.1) | 153 (86.9) |  | | 1.28 (0.57;2.86) | 0.55 | |
| **Mother** |  |  |  | |  |  | |
| Age (years) | 26.6 (22.6;31.5) | 28.1 (24.1;32.1) | 0.21 | |  |  | |
| Parity | 2.5 (± 1.7) | 3.0 (± 1.7) | 0.01 | |  |  | |
| Pre-delivery CD4 counts  (cells per µL) | 490 (426;669) | 522 (436;658) | 0.44 | |  |  | |
| Plasma HIV-1 RNA |  |  |  | |  |  | |
| Undetectable | 36 (40.4)¤ | 219 (48.3)£ | 0.17 | |  |  | |
| Median (log_10_ copies/mL) | 3.31 (2.92;3.98) | 3.41 (2.95;3.90) | 0.70 | |  |  | |
| WHO clinical HIV-1 stage |  |  | 1.00 | |  |  | |
| 1 | 87 (96.7) | 443 (95.7) |  | |  |  | |
| 2 | 3 (3.3) | 19 (4.1) |  | |  |  | |
| 3 | 0 (0.00) | 1 (0.2) |  | |  |  | |
| PMTCT regimen |  |  |  | |  |  | |
| During pregnancy | 86 (95.6) | 443 (95.7) | 1.00 | |  |  | |
| During labour | 85 (94.4) | 454 (98.1) | 0.06 | |  |  | |
| Highest education level completed |  |  | 0.21 | |  |  | |
| None | 14 (15.6) | 47 (10.2) |  | | Ref. |  | |
| Primary | 27 (30.00) | 172 (37.1) |  | | 1.38 (0.58;3.28) | 0.46 | |
| Secondary or tertiary | 49 (54.4) | 244 (52.7) |  | | 2.98 (1.21;7.34) | 0.02 | |
| **Infant** |  |  |  | |  |  | |
| Sex |  |  |  | |  |  | |
| Boys | 47 (52.2) | 229 (49.5) | 0.66 | |  |  | |
| Girls | 43 (47.8) | 234 (50.5) |  | |  |  | |
| Birthweight (g) | 3,100 (2,720;3,400) | 3,000 (2,800;3,380) | 0.78 | |  |  | |
| Breastfeeding duration (weeks) | 40.4 (25.1;45.6) | 40.9 (35.0;45.3) | 0.32 | | 1.02 (1.0;1.04) | 0.09 | |

Values are median (interquartile range) for continuous variable and no. (percentage) for categorical variable.

^a^P value for chi-square or Wilcoxon Mann-Whitney tests.

^b^Variables associated with the outcomes in bivariable regression analysis with a p<0.25 were included in a multivariable regression model. A backward procedure was used to select the final model.
